# Chimpanzees Predict the Hedonic Outcome of Novel Taste Combinations: The Evolutionary Origins of Affective Forecasting

**DOI:** 10.3389/fpsyg.2020.549193

**Published:** 2020-10-06

**Authors:** Gabriela-Alina Sauciuc, Tomas Persson

**Affiliations:** Cognitive Science, Department of Philosophy, Lund University, Lund, Sweden

**Keywords:** affective forecasting, decision-making, episodic system, hedonic predictions, taste stimuli, evolution of cooking

## Abstract

Affective forecasting–predicting the emotional outcome of never-before experienced situations–is pervasive in our lives. When facing novel situations, we can quickly integrate bits and pieces of prior experiences to envisage possible scenarios and their outcomes, and what these might feel like. Such affective glimpses of the future often steer the decisions we make. By enabling principled decision-making in novel situations, affective forecasting confers the important adaptive advantage of eluding the potentially costly consequences of tackling such situations by trial-and-error. Affective forecasting has been hypothesized as uniquely human, yet, in a recent study we found suggestive evidence of this ability in an orangutan. To test non-verbal subjects, we capitalized on culinary examples of affective forecasting and devised a behavioral test that required the subjects to make predictions about novel juice mixes produced from familiar ingredients. In the present study, we administered the same task to two chimpanzees and found that their performance was comparable to that of the previously tested orangutan and 10 humans, who served as a comparison group. To improve the comparability of human and animal performance, in the present study we also introduced a new approach to assessing if the subjects’ performance was indicative of affective forecasting, which relies exclusively on behavioral data. The results of the study open for the possibility that affective forecasting has evolved in the common ancestor of the great apes, providing Hominids with the adaptive advantage of e.g., quickly evaluating heterogeneous food patches using hedonic prediction.

## Introduction

Whether pondering if to try out a new dish, or where to overnight during a long trip, the challenge of making decisions in novel situations is common in our daily lives. To tackle the uncertainty that surrounds such decisions, and to choose efficiently in never-before experienced situations, humans rely on affective forecasting (henceforth AF). AF is conceptualized as a form of affect heuristic, whereby decisions concerning novel situations are based on affective cues triggered in the here-and-now, while simulating^1^ those situations (Gilbert et al., 2002). By bringing the forthcoming into the present, affective forecasts operate by default in an atemporal manner, and have immediate affective effects that motivate choices irrespective of whether these are set to happen in the immediate or more distant future (Gilbert et al., 2002). As such, these affective hints triggered by the choices at hand confer the important adaptive advantage of enabling principled decision-making in never-before experienced situations, thus eluding the potentially costly consequences of tackling such situations by trial-and-error (Gilbert and Wilson, 2007).

Research shows that people are generally adept at predicting the hedonic valence of novel situations (i.e., whether these would feel good or bad), but they systematically overestimate the intensity and duration of such predicted emotions (e.g., Wilson and Gilbert, 2003). Moreover, given the atemporality of AF, prediction accuracy decreases with temporal distance, being high for events expected to happen in the near future, but low for events set to take place in the more distant future (Gilbert et al., 2002). Consequently, much AF research focuses on investigating forecasting biases and strategies to mitigate them, or their applied relevance to decision-making and planning in fields as diverse as economics, law, healthcare, or ethics.

Mechanistically, AF has been theorized to rely on episodic simulation (Gilbert and Wilson, 2007), i.e., on a process that enables people to flexibly integrate information from separate past experiences into novel stimuli or situations (for a recent review see e.g., Schacter et al., 2017). The crucial feature of AF is that affective valuation is engaged during episodic simulation, thus providing decision-makers with hints about the hedonic outcome of a potential course of action (Gilbert and Wilson, 2007). Recent neuroimaging evidence supports this proposal, showing that the so-called default mode network–which is commonly associated with episodic processing (Andrews-Hanna, 2012; Andrews-Hanna et al., 2014; Schacter et al., 2015)–is also involved in AF. More specifically, neural assemblies within the ventromedial prefrontal cortex (vmPFC) underpin memory integration, and link it with associated affective responses to represent novel stimuli and their emergent affective quality (Shenhav et al., 2012; Barron et al., 2013; Benoit et al., 2014). Consistent with early theories that the core processes of AF (i.e., mental construction and hedonic prediction) can take both implicit or explicit forms (Gilbert et al., 1998), the vmPFC–along with the dorsomedial PFC, the hippocampus and the posterior cingulate cortex (PCC)–is found to be implicated in AF regardless of whether the mental construction process is *explicitly* attended to (e.g., when people imagine the taste of novel food combinations), or is used in a valuation process, i.e., when participants are instructed to choose between never-before experienced food combinations (Barron et al., 2013). The medial PFC, the hippocampus and the PCC are key hubs of the default mode network, which, in humans, have been primarily linked to processing information regarding the self, including the simulation of goals and upcoming events (Andrews-Hanna et al., 2014).

In spite of the adaptive significance of AF, research on its evolutionary origins is nearly non-existent. Indeed, AF has been posited as a uniquely human ability, and all animals but humans have been hypothesized to tackle novel situations that are future-oriented by trial-and-error, due to being unable to rely on the flexible information integration characteristic of AF (Gilbert and Wilson, 2007). Recently, however, we found suggestive evidence of AF in a Sumatran orangutan (*Pongo abelii*), who performed successfully in a behavioral test of AF (“the *juice blending task*”) inspired by culinary examples from the AF literature (Sauciuc et al., 2016). For example, given experience with sugar and lemon juice, humans are expected to predict that lemon juice will taste better with sugar than without it even if they never experienced such a mix (Wilson and Gilbert, 2003). When confronted with novel choice situations designed to be similar in structure to this or other similar examples from the AF literature, the orangutan made decisions that reflected hedonic predictions. Moreover, his performance was comparable to that of 10 human participants who served as a comparison group. Not only are humans the only species acknowledged to possess this ability, but brain imaging evidence also shows that humans rely on the episodic system in tasks similar to our *juice blending task* (Barron et al., 2013).

While the results of our previous study suggested the presence of AF in non-human great apes (henceforth apes)–and thus evolutionarily ancient roots for this ability–the conclusions that can be drawn from a single-species, single-subject study are limited. Therefore, the aim of the present study was to test additional non-human subjects, and investigate the presence of AF in another non-human species–the chimpanzee (*Pan troglodytes*). Since AF is a mechanism that supports many forms of human planning (Gerlach et al., 2014; Szpunar et al., 2014), and both observational and experimental studies indicate the presence of planning abilities in chimpanzees (for recent reviews see Osvath and Martin-Ordas, 2014; Scarf et al., 2014; Thom and Clayton, 2016; Trapanese et al., 2019), we hypothesized that chimpanzees will be successful in the *juice blending task*, thus exhibiting behavior consistent with the presence of AF abilities.

This hypothesis is consistent with evolutionary theories that link the emergence of the episodic system to adaptive settings characterized by uncertainty and complexity, where unexpected problems arise frequently and cannot be predicted and prepared for by relying on rigid stimulus-response contingencies acquired by trial-and-error (e.g., Allen and Fortin, 2013). Such types of adaptive challenges are constantly encountered by chimpanzees, given their large dietary repertoire, and their strong affinity for ripe fruit, which is ephemeral and patchily distributed (Potts, 2004; Russon and Begun, 2004). To tackle the spatio-temporal complexity of ripe fruit distribution, chimpanzees rely on a combination of strategies, including enhanced memory, representation and valuation of distant places, resource prediction, route planning, timing of travel, and communication with conspecifics (Potts, 2004; Russon and Begun, 2004; Janmaat et al., 2016). Observational and experimental studies show that the foraging decisions of the great apes, in general, are best predicted by multi-level measures of net benefit, whereby several types of information reflecting both costs and benefits are weighted and integrated. This information spans not only food preferences, but also food quantity, food chemistry (energy content, presence of deterrent substances), processing effort, the energetic costs and time needed to reach a food source, expected level of competition, the presence of predators and other dangers, or the risk of dehydration (e.g., Leighton, 1993; Beran et al., 2009; Janmaat et al., 2014, 2016; Lindshield, 2014; Sánchez-Amaro et al., 2016; Pruetz and Herzog, 2017).

Brain imaging data also shows that the chimpanzees exhibit the relevant neural structures that implement AF in humans. More specifically, the default mode network in chimpanzees appears to be anatomically and functionally similar to that of humans (Rilling et al., 2007; Barks et al., 2015). Compared to humans, however, the chimpanzees exhibit higher activity in the vmPFC (which in humans supports mental construction and the affective valuation of novel situations) and lower activation in the left-sided cortical areas, which in humans are implicated in language and conceptual processing. Rilling et al. (2007) speculated therefore that wakeful rest in the chimpanzees might involve emotionally laden episodic simulation that is independent of language and conceptual processing. Interestingly, and consistent with the hypothesis of Allen and Fortin (2013) on the evolutionary pressures that prompted the emergence of the episodic system, the size of the vmPFC in the primate lineage increases significantly with dietary spectrum and complexity of foraging strategies (Louail et al., 2019).

In conclusion, behavioral and neural evidence converges toward the hypothesis that AF should be part of the chimpanzee cognitive repertoire. Cumulative evidence shows that chimpanzees retain and recall a variety of characteristics of food items and feeding locations, and they flexibly access these memories for the purposes of decision-making, even when foods–and the potential benefits or risks associated with these–are outside direct perceptual range. Flexible memory integration and valuation in order to take the best course of action are the key cognitive operations implicated in AF (Gilbert and Wilson, 2007; Barron et al., 2013). Moreover, the neural structures that are crucially implicated in human AF exhibit functional similarities in chimpanzees, and, evolutionarily, appear subjected to adaptive pressures derived from foraging complexity.

Finally, a second aim of this study was to develop and evaluate a novel approach to AF assessment, in order to simplify the procedure and increase the comparability of human and non-human performance in the juice blending task. In our previous study we compared *test* preference measures (based on behavioral responses) to *post-test* preference measures that differed across species, i.e., self-reports for humans and behavioral responses for the orangutan. In the present study we use exclusively behavioral measures, and compare *test* preferences to a predictive model of preferences for the novel *mixes*, which is derived from *pre-test* preferences for *ingredient* juices.

## Materials and Methods

### Subjects and Site

Two chimpanzees housed at Furuvik Zoo/Lund University Primate Research Station Furuvik (Sweden)–one male (Tjobbe, born 2003, transferred to Furuvik in 2015 from Veszprém Zoo, Hungary) and one female (Maria Magdalena, born 2000, transferred to Furuvik in 2005 from Borås Zoo, Sweden)–completed the study. Both chimpanzees were mother-raised in captivity. Three additional chimpanzees from the same facility did not pass the *pre-test* phase and were excluded from further testing. With the exception of Tjobbe, all chimpanzees had previously participated in studies targeting cognitive abilities. None of these, however, focused on AF. At the time of testing the chimpanzees were housed in two groups, with Maria Magdalena and Tjobbe being part of the same group. The chimpanzees are fed a varied diet that includes vegetables, fruits, seeds, protein sources and enriched pellets, with vegetables and pellets being the staple components of the diet. To limit intake of soluble sugars, fruit is only served in small quantities, usually as a reinforcer during husbandry training. The main feedings of the day occurred at 08.00, 12.00, and 15.00. All testing sessions with the chimpanzees were conducted between 10 and 11.30, and between 13.30 and 14.30.

### Procedure

### General Procedure

The procedure was similar to the one in Sauciuc et al. (2016), except that: (1) the chimpanzees received choices between equal stimulus portions in the test phase; (2) AF assessment was conducted by comparing choices in the test phase to a predictive model derived from pre-test preferences for the ingredients (as detailed further below).

Throughout the study, the subjects were presented with binary choices between two juices. Depending on study phase, the two juices were either two *ingredient* juices (*pre-test* phase) or a pair formed by a familiar *ingredient* and a novel *mix* (*test* phase). Most juices were derived from commercially available concentrates, by diluting them with water. These juices were specifically selected by the researchers to be different from the juices occasionally consumed by the chimpanzees at the zoo. Whenever available, organic products were used. To increase juice distinctiveness, juices with similar color (e.g., lime, lemon, apple, pineapple) were dyed using food-grade dyes. The juices were presented in 10 ml portions, in small transparent bottles. At the start of each trial, choice bottles were briefly presented on a table at a distance of approximately 30 cm from the chimpanzees, who were sitting behind a polycarbonate window. The bottles were then pushed toward two (of three) holes in the window so that the chimpanzees could make a choice (as exemplified in the film included in Supplementary Video 1). The chimpanzees accessed the chosen liquid with the help of a drinking straw or by having it poured into their mouth by the experimenter, depending on whether the subject could use straws for drinking. All experimental sessions were video-recorded.

Data collection took place between September 2015–December 2016, involving two experimenters: one who prepared the stimuli, and one who administered the trials. The presence of two experimenters was required in order to ensure good presentation flow, and thus keep the subjects focused and motivated. In spite of these efforts, the chimpanzees appeared less focused and more easily distracted compared to the orangutan tested in Sauciuc et al. (2016). As a consequence, several testing sessions included unplanned breaks.

It is worth noting that, before conducting data collection for the purposes of Sauciuc et al. (2016), several aspects of the procedure were pilot tested with additional human participants (i.e., participants who are not part of the final sample used in Sauciuc et al., 2016). The first pilot tests were focused on aspects of trial administration and stimulus preparation, including establishing a suitable concentration for the less pleasant items (which were diluted with water), establishing a suitable portion size, choosing suitable containers, or determining if individuals experienced the juices differently when consumed by using a straw as opposed to drinking directly from the container. Subsequently, more encompassing pilot testing was carried out with a focus on potential interferences that could affect the subjects’ choices, in particular interferences that could lead to choices that contradicted AF. To be able to tap into such interferences, we administered a large number of trials, that spanned all experimental phases. Pilot test participants were also asked to verbalize as much as possible throughout the testing. Pilot testing revealed, for example, that participants may make odd choices for a variety of reasons, including curiosity, suspecting deception, distraction, interference from food items consumed prior to testing, or momentary preference fluctuations. Pilot testing also allowed us to observe choice outcomes and patterns when participants used other strategies than AF, such as random choice, or sampling only the mixes in order to learn them.

### Pre-test Phase: Stimulus Selection and Ingredient Preference Ranking

The aim of this phase was to establish a set of four distinctly colored and distinctly flavored *ingredient* juices for which a stable preference ranking could be determined. To this end, binary choices between two juices were repeatedly presented (see Supplementary Video 1 for examples) until the subject showed a clear preference for one of the two juices, i.e., the subject chose one juice over the other in five of six consecutive trials.

When a subject appeared to have formed stable preferences for a set of four *ingredients*, 24 additional trials (four trials × six ingredient pairs) were administered, in which the *ingredient* pairs were presented in a randomized order rather than in blocked trials as previously. A final *ingredient* preference ranking (henceforth *pre-test ranking*) was then established for each subject based on how often the subject chose each *ingredient* in these 24 randomized trials. Additional consolidation trials were administered when several weeks had passed between *pre-test* sessions or preferences appeared to change. If preference changes persisted, the involved *ingredients* were replaced, and the procedure was repeated from the beginning.

The total number of trials received by each subject in the *pre-test* phase varied, depending on how quickly each individual *ingredient set* could be established. Before moving to the test phase, Tjobbe received 225 *pre-test* trials (including preference consolidation trials), and his *ingredient set* included, in the order of *pre-test ranking*: pomegranate > pineapple > golden grapefruit > lemon juices. Maria Magdalena was tested twice, and graduated to the *test* phase after receiving a total of 255 trials and 81 trials, respectively. Her *ingredient sets*, in the order of *pre-test ranking*, included: cherry > orange > apple > lemon juices (set 1), and cherry > pineapple > pink grapefruit > lime juices (set 2). The three chimpanzees that did not advance to the *test* phase received between 164 and 746 *pre-test* trials before being excluded from the experiment.

### The AF Test Phase

To examine the subjects’ ability to make predictions concerning the hedonic outcome of never-before experienced situations, we presented them with novel choice situations inspired by culinary examples from the AF literature, in which they had to choose between a *familiar ingredient* and a *novel juice mix*. The latter was created in front of the subject, by combining two familiar *ingredients* (see Supplementary Video 1 for examples). Six novel juice *mixes* and 24 never-before experienced choice situations were derived by systematically applying this procedure. The choice situations were presented in randomized order, and occurred only once in a block of 24 trials. To prevent the possibility that the subjects learned the optimal outcome of each choice situation through associative learning, the *test* phase consisted of only two blocks of trials (in total 48 trials). In other words, each of the 24 unique choice situations occurred only twice, with the second presentation occurring only after all the other 23 choice situations were presented once.

While in Sauciuc et al. (2016), each *test* trial consisted of a choice between 10 ml of a familiar ingredient and 20 ml of a novel mix (obtained by combining two ingredient portions of 10 ml each), in the present study, instead, each *test* trial offered two liquids of equal volume, with portion volume being initially set at 10 ml. The first tested subject (Maria Magdalena) received initially *test* trials consisting of choices between 10 ml of ingredient and 10 ml of *mix*, the latter being produced by combining two *ingredient* portions of 5 ml each. Such a small volume, however, raised concerns regarding good stimulus visibility. To correct this issue, portion volume was set at 20 ml when Maria Magdalena was tested a second time, with a different *ingredient set*. Tjobbe received only the second version of the test.

### Assessment of AF

To determine if participants relied on AF in the test trials, in Sauciuc et al. (2016) correlation analyses were conducted to compare choice-derived preferences from the *test* trials with *post-test* preference measures. The latter were obtained by self-report (humans) or from additional choice trials in which *ingredients* and pre-blended *mixes* were “disguised” by new colors and administered in preference tests (the orangutan). Since self-reports were collected shortly after task completion, they could have been biased by memories of recently made choices rather than reflecting a hedonic ranking for ingredients and mixes.

In the present study, instead, choice-derived preferences from the 48 test trials (henceforth *test ranking*) were compared with a predictive model of preference ranking for *ingredients* and *mixes*, henceforth *predicted ranking*. This was derived from the *pre-test ranking* for the *ingredients*, whereby each *ingredient* was first assigned a rank from 1 (least preferred *ingredient*) to 4 (most preferred *ingredient*). Reasoning that *ingredients* are downgraded or upgraded to various degrees when mixed with other *ingredients*, we calculated a predicted score for each *mix* based on the formula: rank _*mix*_ = (rank_*ingredient 1*_ + rank_*ingredient 2*_)/2. (Please note that this is a simple mathematical formalization reflecting the upgrading or downgrading of *ingredient* value when mixed with other *ingredients*, and not a claim that the participants–human or non-human–explicitly used this formula when making optimal choices in the test trials). The predicted scores can thus be used both for deriving a *predicted ranking*, and for predicting the expected outcome of each novel choice situation. For example, if cherry is the most preferred *ingredient* (score 4) and rhubarb the next preferred one (score 3), this allows to predict that a *mix* of cherry and rhubarb (with a derived score of 3.5) should be less preferred than (and thus not chosen over) cherry juice but more preferred than (and thus chosen over) rhubarb juice. In turn, this allows us to assess if the subject’s choices are indicative of AF, by comparing predicted choice outcomes (henceforth *predicted choices*) with actually observed choice outcomes (henceforth *actual choices*).

### Ethical Note

All applicable international, national and/or institutional guidelines for the care and use of great apes were followed. All procedures were in accordance with the ethical standards set by the Swedish Board of Agriculture concerning non-invasive research with apes. In accordance with the latter, the experimental protocols for the experiment were approved by the Regional Ethical Review Board at Uppsala District Court (permit C110/15).

## Results

### Predicted Ranking Validation

To establish the validity of the new approach to AF assessment, we applied this approach to the data obtained in Sauciuc et al. (2016), in which we found evidence of AF when 10 human participants and an orangutan were tested with the *juice blending task*. To this end, we assessed if the *predicted ranking* for the ingredients and mixes (which is derived as described further above, in the “Materials and Methods” section) had a similar distribution to the previously used measures, i.e., *test ranking* and *post-test* ranking. To this end, non-parametric ANOVAs were conducted for each data set obtained in Sauciuc et al. (2016). For all 11 datasets the results (see Table 1, column 3) were non-significant, ranging between χ^2^(2) = 1.514 (*p* = 0.495) and χ^2^(2) = 0.51 (*p* = 1). Thus, the null hypothesis that the three measures had similar distribution (i.e., the ranking of the 10 ingredients and mixes did not significantly differ between *predicted*, *test* and *post-test ranking*) could not be refuted.

**TABLE 1:** Overview of results for the statistical analyses conducted in the study.

Species*	**Dataset**	**Friedman’s ANOVA (χ^2^)**	**Kendall’s tau-b: test and predicted ranking**	**Kendall’s tau-b: predicted ranking and post-test ranking**	Predicted vs. actual choices**
Human	P1	0.378, *p* = 0.872	0.800, *p* = 0.002	0.629, *p* = 0.014	87%, *p* < 0.001
Human	P2	0.171, *p* = 0.949	0.738, *p* = 0.005	0.874, *p* = 0.001	85%, *p* < 0.001
Human	P3	0.154, *p* = 0.947	0.610, *p* = 0.021	0.782, *p* = 0.002	85%, *p* < 0.001
Human	P4	0.211, *p* = 0.916	0.714, *p* = 0.006	0.644, *p* = 0.011	81%, *p* < 0.001
Human	P5	0.051, *p* = 1.000	0.708, *p* = 0.007	0.690, *p* = 0.007	90%, *p* < 0.001
Human	P6	0.359, *p* = 0.897	0.726, *p* = 0.007	0.414, *p* = 0.103	81%, *p* < 0.001
Human	P7	0.054, *p* = 0.992	0.833, *p* = 0.001	0.828, *p* = 0.001	94%, *p* < 0.001
Human	P8	0.051, *p* = 1.000	0.800, *p* = 0.002	0.908, *p* < 0.001	90%, *p* < 0.001
Human	P9	0.053, *p* = 0.989	0.771, *p* = 0.003	0.736, *p* = 0.004	88%, *p* < 0.001
Human	P10	0.158, *p* = 0.956	0.659, *p* = 0.012	0.552, *p* = 0.030	77%, *p* < 0.001
Orangutan	Naong	1.514, *p* = 0.495	0.833, *p* = 0.001	0.643, *p* = 0.013	94%, *p* < 0.001
Chimpanzee	M. Magdalena 1	N/A	0.578, *p* = 0.027	N/A	85%, *p* < 0.001
Chimpanzee	M. Magdalena 2	N/A	0.565, *p* = 0.029	N/A	79%, *p* < 0.001
Chimpanzee	Tjobbe	N/A	0.930, *p* < 0.001	N/A	88%, *p* < 0.001

*Statistical analyses for the human participants and the orangutan are based on the data collected in Sauciuc et al. (2016). For the chimpanzees, these analyses are based on the data collected in the present study. **p values are based on binomial tests, two-tailed.

Following Sauciuc et al. (2016), Kendall’s tau-b correlations were computed to determine the strength and size of the relationship between *predicted ranking* and the other two ranking measures, i.e., *test ranking* and *post-test ranking*. A total of 22 correlation analyses were thus carried out [i.e., two correlations for each of the 11 datasets obtained in Sauciuc et al. (2016)], and the sample size for each correlation was always *N* = 10, which reflects the number of choice items for which preference rankings were obtained.

For all 11 datasets obtained in Sauciuc et al. (2016), *predicted ranking* correlated significantly with *test ranking*. Correlation coefficients for the human participants (Table 1, column 4) ranged from τ_*b*_ = 0.61 (*p* = 0.02, *N* = 10) to τ_*b*_ = 0.83 (*p* = 0.001, *N* = 10). For the orangutan, the correlation coefficient matched the highest human score: τ_*b*_ = 0.83 (*p* = 0.001, *N* = 10). For nine human participants, the *predicted ranking* correlated significantly with *post-test ranking* (Table 1, column 5), with correlation coefficients ranging from τ_*b*_ = 0.552 (*p* = 0.03, *N* = 10) to τ_*b*_ = 0.908 (*p* < 0.001, *N* = 10). The non-significant correlation obtained for one human participant (P6) was due to changes in the preference ranking for the *ingredients* between *pre-test* and *post-test*. This highlights once more the need for an approach that minimizes effects of repeated exposure. For the orangutan, the correlation between *predicted ranking* and *post-test ranking* was τ_*b*_ = 0.643 (*p* = 0.013, *N* = 10).

Additional statistical analyses were conducted for assessing the extent to which *actual test choices* reflected *predicted choices*. For the human participants as a group, *actual choices* matched *predicted choices* in 85% of the trials, i.e., for an average of 40.9 trials [SD = 2.558; *Min* = 37 trials (77%); *Max* = 45 trials (94%), see Table 1, column 6 for more details]. For the orangutan, *actual choices* matched *predicted choices* in 94% (*N* = 45) of the 48 test trials. Binomial tests conducted individually for each dataset revealed that performance was significantly above chance levels (all *p*-s < 0.001) for all the humans and the orangutan.

### AF in Chimpanzees

To determine if the chimpanzees’ choices in the *test* trials were guided by AF, we compared *predicted* and *test ranking* (see Supplementary Material 1 for individual rankings). The two measures were significantly correlated for all datasets: τ_*b*_ = 0.578, *p* = 0.027, *N* = 10 (Maria Magdalena, *ingredient set* 1); τ_*b*_ = 0.565, *p* = 0.029, *N* = 10 (Maria Magdalena, *ingredient set* 2); τ_*b*_ = 0.93, *p* < 0.001, *N* = 10 (Tjobbe). Compared to the previously tested participants, the chimpanzees’ scores fell at the two extremes of the human range, with the female’s scores being the lowest and the male’s score being the highest.

We further compared the chimpanzees’ *actual test choices* with *predicted choices* and found them to match in 85% of the *test* trials, i.e., for an average of 40.7 trials [SD = 2.517, *Min* = 38 (79%), *Max* = 42 (88%)]. Binomial tests revealed that, for all three chimpanzee datasets, performance was significantly above chance levels (all *p*-s < 0.001), thus suggesting that their performance did not reflect a trial-and-error approach to the novel choice situations.

**FIGURE 1:**
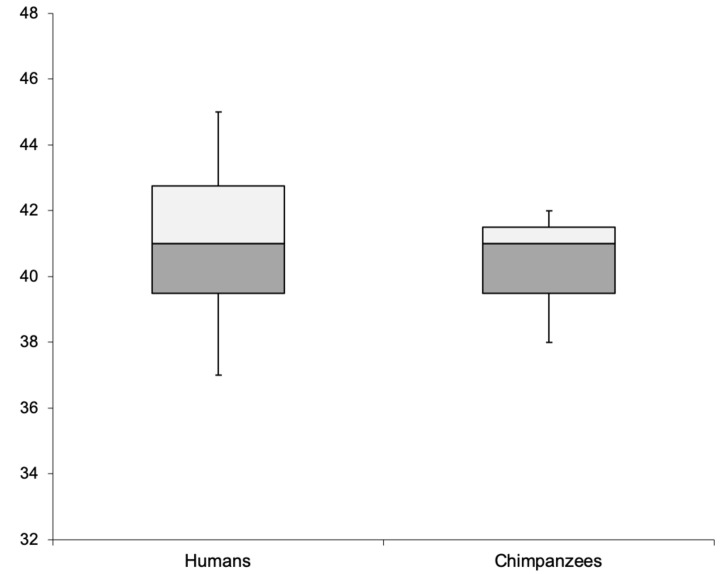
Comparison of the human and chimpanzee performance with respect to the number of *actual choices* that matched *predicted choices* in the test trials.

To compare task performance between chimpanzees and the previously tested human participants, we conducted a Mann–Whitney *U* test with tie correction. No significant statistical differences were detected between the performance of the chimpanzees and that of the human participants: *U* = 16, *p* = 0.930, *Mdn*_*Hum*_ = 41, *Mdn*_*Chimp*_ = 41 (see also Figure 1).

### Additional Analyses for Discounting Alternative Strategies to AF

In this section, we report a number of additional data analyses which were conducted to rule out that the chimpanzees’ choices in the test trials could be explained by alternative strategies to AF. We first tested if the choices made by the chimpanzees in the test trials were affected by a negative bias toward the novel *mixes*. Such a bias would be expected if the subjects did not understand how *ingredients* were transformed when mixed with other *ingredients*, being thus unable to predict the hedonic value of the *mixes*. In the 48 test trials, the chimpanzees chose on average 21.33 *ingredients* and 26.66 *mixes*, and were not more likely to choose familiar *ingredients* over novel *mixes* (all *p*-s ≥ 0.111, binomial test, two-tailed). These results rule out the presence of a negative bias toward the *mixes*. Moreover, separate comparisons between each chimpanzee dataset and each previous participant showed that their *mix*-to-*ingredient* ratio did not differ significantly (all *p*-s > 0.05, Fisher’s exact test) from that of the previously tested orangutan (21 ingredients vs. 27 mixes) or humans (23.5 ingredients vs. 24.5 mixes).

**FIGURE 2:**
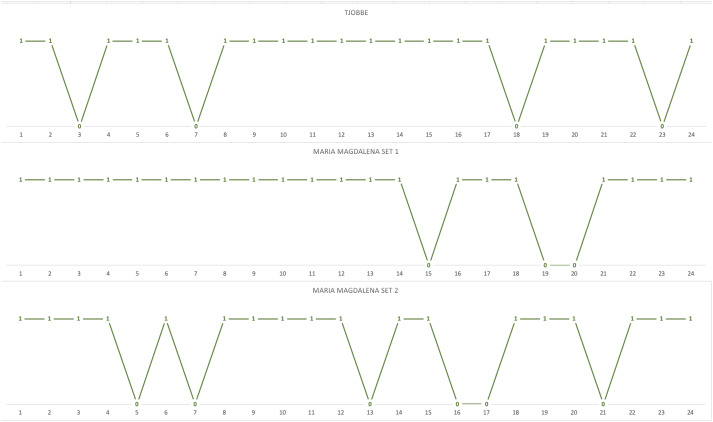
Trial-by-trial distribution of the chimpanzees’ *actual choices* that matched *predicted choices* in the first block of 24 unique trials (1 = matching choice; 0 = non-matching choices).

To follow-up on potential learning effects, block-by-block comparisons were also conducted. The chimpanzees’ *actual choices* matched *predicted choices* for an average of 20 choices (82%) in the first block of trials, and for an average of 21 choices (88%) in the second block of trials. The results of a Mann–Whitney *U* test with tie correction suggested no significant improvement in the first block of trials compared to the second: *U* = 3, *p* = 0.643, *Mdn*_*block 1*_ = 20, *Mdn*_*block 2*_ = 20. No significant differences were detected at individual level in the performance of each chimpanzee in the first and second trial block (all *p*-s > 0.05 Fisher’s exact test). Taken together, these results do not support the alternative hypothesis that learning affected the chimpanzees’ performance. This is further confirmed when inspecting the trial-by-trial distribution of *actual choices* that matched *predicted choices* in the first block of 24 trials, i.e., as the chimpanzees were confronted for the first time with each of the 24 novel choice situations. Indeed, there is no clustering of mismatches within the first part of the block, nor is there an increase in the number of matches toward its end (see Figure 2). Since, for both ingredient sets, Maria-Magdalena received the first block of test trials in an afternoon session and the second block of test trials in a morning session, and given the lack of significant differences in performance across the two blocks of test trials, this also suggests that time of day influences on task performance are unlikely.

Additional analyses were conducted to rule out that the chimpanzees did not employ an ingredient-tracking strategy, whereby they systematically pursued the most preferred item, or avoided the least preferred item disregarding the other ingredient in a mix or the other item in the choice. Since, for some trial types, this strategy and AF make similar predictions, these analyses focused on two trial types (henceforth “key trials”) for which the two strategies predict diverging outcomes (for more details, see the datasets in Supplementary Material 1). The first key trial type, which allows discounting that participants solve the task by tracking the most preferred item, contrasts 20 ml of the second most preferred ingredient (rank 3) with a mix of 10 ml of the most preferred ingredient (ranked 4) and 10 ml of the item ranked 1. For this trial type, choosing the ingredient is the outcome predicted by AF, while choosing the mix is the outcome predicted by tracking the most preferred item. The second key trial type allows discounting that the participants solve the task by avoiding the least preferred ingredient, and contrasts 20 ml of the item ranked 2 and a mix of 10 ml of the least preferred ingredient (ranked 1) and 10 ml of the ingredient ranked 4. For this trial type, the outcome predicted by AF is choosing the mix, while the outcome predicted by avoiding the least preferred item is choosing the ingredient.

We found that the choices of the male chimpanzees conformed 100% to the predictions made by the AF strategy, while the choices made by the female chimpanzees conformed 50% to the predictions made by the AF strategy. Overall, the chimpanzees’ choices in the key trials were consistent with an AF strategy in 67% of the trials (i.e., 8 of 12 trials). For comparison, 63% of the choices made by the human participants in the key trials were consistent with an AF strategy (i.e., 25 of 40 trials). Only one of the human participants had a perfect score, similarly to the male chimpanzee. Four of the human participants performed similarly to the female chimpanzee, i.e., 50% of their choices were consistent with AF predictions and one performed worse than the female chimpanzee. The choices of the remaining human participants were 75% consistent with AF predictions. The previously tested orangutan was the third participant with a perfect score, besides the male chimpanzee and one of the human participants. A cross-species comparison on the distribution of AF-consistent choices showed that the performance of the chimpanzees in the key trials did not differ significantly from that of the human participants (χ^2^ = 0.069, *p* = 0.793). This did not change when the orangutan score was cumulated with the score of the chimpanzees to compare the performance of non-human apes with that of humans: χ^2^ = 0.797, *p* = 0.372.

Since our sample size consisted of only two chimpanzees, and since the performance of the two chimpanzees matched the highest and the second lowest human range–i.e., 100% consistent and 50% consistent, respectively–additional analyses were carried out to determine if the distribution of the highest and lowest scores among the human participants would suggest superior performance by the humans. This comparison indicated that a perfect score did not occur more frequently among humans than chimpanzees (χ^2^ = 0.965, *p*-value = 0.326), and that the low score of 50% consistent with AF was not more infrequent in humans compared to chimpanzees (χ^2^ = 0.444, *p* = 0.505). The results remained non-significant when the orangutan and chimpanzee data was pooled, although there was a (non-significant) trend toward better performance in apes vs. humans (perfect score distribution: χ^2^ = 2.715, *p* = 0.099; low score distribution: χ^2^ = 0.034, *p* = 0.853). (Note that the human participant who performed worse than the female chimpanzee was excluded from the analyses on the distribution of the 50% score).

Additional relevant trials for ruling out the use of an ingredient-tracking strategy are the trials for which the AF model predicts a tied outcome (henceforth “tie-trials”). In these trials, the best fitting outcome is switching between choosing the ingredient and the mix across the two encounters with the same tie-trial. Exhibiting a bias toward the mix or ingredient in both encounters with a given tie-trial cannot be judged as incorrect either, although it can be indicative of biases concerning specific ingredients, such as pursuing the most preferred ingredient or avoiding the least preferred one. Throughout the test phase, an individual encounters four such tie-trials (i.e., two trial types × two occurrences, for more details, see the datasets in Supplementary Material 1).

We first carried out analyses to determine the frequency of the switch vs. biased outcomes in each species. We found that the “switch” approach (which is the one that best conforms to the predictive model), was used by the chimpanzee male (once), by the orangutan (once), and by two human participants. Of the two humans, one adopted the switch for both tie-trial types. The switch strategy thus accounted for 17% of the trials in chimpanzees and 15% of the trials in humans. If the orangutan data is cumulated with that of the chimpanzees, then the apes’ score increases to 25%. Analyses carried out on the distribution of switch vs. biased outcomes in the choices made by each species revealed no significant difference between humans and chimpanzees (χ^2^ = 0.01, *p* = 0.921), or between apes and humans (χ^2^ = 0.390, *p* = 0.533).

For the tie-trials in which the individuals exhibited a biased outcome, by choosing the ingredient or the mix, we assessed if the distribution of the outcomes (ingredient, mix) differed between species. We found that there was no significant difference between chimpanzees and humans in this respect (χ^2^ = 0.528, *p* = 0.467), or between apes, in general, and humans (χ^2^ = 1.468, *p* = 0.226). More specifically, the humans exhibited an ingredient bias in 76% of the trials (13 of 17 trials), while the chimpanzees showed a similar bias in 60% of the trials (3 of 5 trials). A closer look at these trials revealed that the humans showed a higher tendency than the chimpanzees to avoid mixes that contained the least preferred ingredient. In chimpanzees, this tendency was present in 1 out of 5 trials (20%), while in humans it was present in 8 out of 17 trials (47%). However, no significant difference was found between the chimpanzees and humans in this respect: χ^2^ = 1.170, *p* = 0.279 (If the orangutan data is added, the results are updated to 1 out of 6 trials in apes (17%), and χ^2^ = 1.720, *p* = 0.190).

Additional analyses revealed that this trend was not driven by outliers among the human subjects, as the tendency to avoid a mix containing the least preferred ingredient was present in eight of the ten human datasets. This tendency was present in one of the three chimpanzee datasets (or one of four ape datasets). The comparison between humans and chimpanzees was non significant: χ^2^ = 2.360, *p* = 0.125. The cross-species comparison between apes and humans on the distribution of the tendency to avoid mixes containing the least preferred ingredient approached significance–χ^2^ = 3.764, *p* = 0.052.

The converse strategy of tracking the most preferred ingredient was potentially present in one chimpanzee trial (i.e., 20% of the relevant chimpanzee trials) and in three human trials (18% of 17 relevant trials). If the orangutan data is included, the ape percentage decreases to 17% (i.e., one of six relevant ape trials). The cross-species comparison on the distribution of this potential bias showed no significant difference between chimpanzees and humans (χ^2^ = 0.014, *p* = 0.905), or between apes and humans (χ^2^ = 0.003, *p* = 0.957). In addition, the between-species comparison was non-significant when the distribution of this potential bias was assessed at the level of individual datasets: χ^2^ = 0.012, *p* = 0.913 (χ^2^ = 0.035, *p* = 0.852 if the orangutan data is included).

Overall, the results reported in this section reveal that the similarity of performance between chimpanzees and human participants runs down to the details (strategies, biases) of how different types of trials are approached. These results are inconsistent with an interpretation that the participants’ choices were driven by alternative strategies to AF. Instead, the predictive model based on hedonic predictions offers the best explanatory account for the choice patterns observed in the test trials.

## Discussion

Affective forecasting (AF) is pervasive in our lives, steering decisions and planning. To determine if chimpanzees exhibit this ability, in this study we administered a *juice blending task*, which was originally devised for testing AF in an orangutan (Sauciuc et al., 2016). Two chimpanzees graduated to the *test* phase, and both successfully solved the task. Moreover, their performance was comparable to that of previously tested subjects–a Sumatran orangutan and 10 humans. Inspired by culinary examples from the AF literature, the task required the subjects to integrate information about familiar juices, and to predict the hedonic outcome of the never-before experienced juice *mixes*. The latter were presented in novel choice situations, i.e., 24 unique pairings between a familiar *ingredient* and a novel *mix*. In order to make optimal choices, the subjects had to be sensitive to the taste and value transformations that the *ingredients* underwent when mixed with other *ingredients*, and make hedonic predictions concerning the value of the novel *mixes* based on the known values of the *ingredients*. In addition, choice efficiency required an ability to derive the relative values of two items that, prior to the *test* phase, have not been paired in a choice, i.e., to compare the hedonic prediction about the *mix* to the known value of the other juice available in the choice. Conversely, the lack of such abilities would lead to suboptimal choice strategies, such as trial-and-error or a familiar-item bias.

To ascertain that subjects’ choices in the novel choice situations reflected AF, we compared them with a predictive model of preference ranking for *ingredients* and *mixes*. Multiple statistical tests were run, and all converged toward the conclusion that, when confronted with never-before experienced *mixes* presented in never-before experienced choice situations, the chimpanzees made efficient choices that reflected hedonic predictions. In addition, the chimpanzees’ performance was comparable to that of human participants tested in a previous study.

We also assessed and ruled out the possibility that the choices made by chimpanzees in the *test* trials could be explained by strategies suggesting a failure to evaluate the *mixes*, such as trial-and-error or a negative bias toward the *mix*. We found that the chimpanzees were not more inclined to choose the familiar *ingredient* over the novel *mix* (nor vice-versa), and their *mix*-to-*ingredient* ratio did not differ from that of the previously tested orangutan and humans. Note that a similar outcome would be also expected if the subjects took a stimulus generalization approach in the *test* trials, as this approach would predict that the subjects should select the stimuli that best approximate “trained” stimuli–in this case the *ingredients*. Moreover, the *actual choices* of the chimpanzees matched *predicted choices* to levels that were significantly above chance. These results falsify the hypothesis of Gilbert and Wilson (2007) that non-human animals approach never-before experienced situations by trial-and-error.

Another potential strategy that is not suggestive of an AF ability, involves tracking particular *ingredients*, such as the most or least preferred ones, in order to always select or avoid them, respectively. Such a strategy, however, would result in choices that are not sensitive to the manipulations, i.e., whether the *ingredients* are upgraded or downgraded when mixed with other *ingredients*. As such, it would be expected that *mixes* that contain the least preferred juice would be systematically avoided irrespective of the other *ingredient* in the mix and irrespective of the other choice item. Conversely, the *mixes* containing the most preferred item should be chosen regardless of the other juice in the *mix* and regardless of the other juice in the choice. Generally, the chimpanzees selected the *mixes* containing the most preferred juices as long as their estimated pleasantness exceeded that of the other item in the choice, and avoided *mixes* containing the least preferred item as long as their estimated pleasantness was lower than the other juice in the choice. In other words, the chimpanzees made optimal choices that reflected an assessment of the relative value of the items (familiar ingredients vs. novel mix) present in the choice.

This was confirmed by several statistical analyses aimed at comparing the performance of chimpanzees and humans in a number of key trials for which AF and ingredient-tracking make contrasting predictions. The performance of the two chimpanzees matched the highest and second lowest scores of the human range, and there was no significant difference between the performance of the two species. Interestingly, the male chimpanzee was one of three individuals who exhibited a perfect score in these trials–the other two individuals being one human participant and the orangutan tested in Sauciuc et al. (2016). Additional statistical analyses conducted separately for trials where AF predicted a tied outcome revealed once more that the chimpanzees’ performance did not differ significantly from that of the humans. Together with two humans and the previously tested orangutan, the male chimpanzee was again among the few individuals who chose the approach that best fitted the outcome predicted by AF.

The analyses carried out on tie-trial performance suggested that the human participants were often inclined to avoid the mix that contained the least preferred ingredient even if this mix had a similar value to the other choice item. This was the only cross-species comparison that approached significance, thus potentially suggesting that the apes were less susceptible to such a bias. We refrain, however, from drawing any further conclusions from these results, given the small samples, and also because we cannot quantify affect intensity for the ingredients and prospective mixes in a way that would allow between-subject comparisons. In other words, it is possible that several human participants experienced their least preferred ingredient as more unpleasant than others did, or than the apes did. In addition, rather than being an indicator of an ingredient-avoidant strategy, the tendency to avoid the mix containing the least preferred ingredient could be interpreted as indicative of a commonly encountered forecasting bias, whereby the intensity of expected negative (or positive) affect is exaggerated.

As reviewed above, the results of this study cannot be explained by alternative choice strategies driven by associative learning-effects. Moreover, the task administered in the *test* phase was specifically designed to prevent associative-learning, which is commonly described as a relatively slow process that initially entails trial-and-error and at least minimal–but most often prolonged–experience with stimuli (e.g., Gilbert and Wilson, 2007; Pearce, 2008; Gershman and Daw, 2017). In order to prevent associative learning, we administered a minimum number of *test* trials, i.e., there were only two trials for each of the 24 unique choice situations, with the second trial being presented only after each of the 24 unique choice situations has been presented once. As such, the first direct experience with the outcome of each novel choice situation occurred only after the completion of a prospective decision-making process that required information integration and predictive valuation. Statistical analyses showed that the chimpanzees exhibited almost identical performances in the first and second blocks of *test* trials. This is not compatible with a learning account because it indicates that the chimpanzees made optimal choices from the very first encounter with the novel choice situations. Since within a block of *test* trials, a *mix* occurred four times, each time paired with a different *ingredient*, it could be argued that this re-occurrence of the *mixes* provided the subjects with direct experience of the novel *mix* as soon as that *mix* has been consumed once. It has to be noted, though, that none of the *mixes* was chosen and consumed on all four occasions in which it occurred. Moreover, given trial randomization, the experience of consuming a given *mix*–whenever a *mix* was chosen–was embedded in a rapid sequence of other gustatory experiences. Thus, the combinatorial demands of the procedure–involving several novel items that occurred randomly in a variety of contexts, and always required new value computations–were likely beyond the explanatory power of associative learning. In addition, brain imaging data shows that, in humans, the demands for *de novo* mental construction do not disappear completely after the first direct experience with a novel food blend, although they diminish considerably (Barron et al., 2013). Consistent with this, our pilot data indicated that a human participant who sampled primarily novel *mixes* with the purpose of learning them succeeded in this endeavor only after 48 test trials (i.e., after completing a first and second trial for each offer type).

Finally, it is important to discuss whether the *juice blending task* is appropriate for assessing AF as a case of episodic simulation. Human brain imaging data shows that the construction and valuation of never-before experienced food mixes engages neural structures that are commonly involved in episodic simulation, regardless of whether this process is attended to explicitly (Barron et al., 2013). Clinical evidence further suggests that the cognitive and affective processing of food experiences are dissociated between the semantic and episodic systems. Food recognition and knowledge about food compatibility is disrupted in patients with semantic dementia. The hedonic evaluation of flavor combinations–which is also targeted in our study–remains intact in such patients, but is impaired in patients with episodic memory deficits (Piwinca-Worms et al., 2010). This evidence indicates that our task indeed taps into the episodic system and AF. As a rule, however, similarity of behavioral performance does not necessarily entail that similar cognitive and neural mechanisms were engaged by the different species when solving this task (for an extensive discussion, see e.g., Leavens et al., 2019). The plausibility of such an interpretation needs to be pondered in the light of evidence from additional studies that investigated the presence in apes of key cognitive (i.e., complex, context-based value computations, memory integration into novel stimuli) and neural mechanisms (i.e., components of the default network) known to be implicated in humans during AF and, indeed, in a task similar to ours.

At least two kinds of complex value computations are relevant here. The first is the ability to flexibly determine the relative value of two familiar items that have never been paired before. This ability has been previously investigated in studies on transitive inference and flexible quality-quantity trade-offs, and its presence has been documented not only in apes, but also monkeys (e.g., Padoa-Schioppa et al., 2006). While this form of flexible value computation involves context novelty, there is no novelty with respect to the choice items, as their values are well-known to the subjects. The second relevant ability–to date only documented in apes–is the kind of complex value discrimination required when computing the relative value of single and compound choice items, such as when choosing between a slice of banana vs. a slice of banana and a slice of apple (Beran et al., 2009; Sánchez-Amaro et al., 2016). This task is somehow reminiscent of the *juice blending task* used in our study, since our task also involves a single and a compound item, and each trial begins with the presentation of three items (i.e., three juice bottles). Thus, this raises the question whether the apes could have solved the *juice blending task* by resorting to this form of complex value discrimination only. We believe this is not likely because, in spite of some superficial resemblance, the demands of the two tasks differ in crucial ways. No visual, nor value transformation is involved in choices between a single and a compound item. Optimal performance in a task that involves compound items that do not undergo transformation arguably requires a simpler process of summation. In its simplest form, such as in the example given above, this boils down to recognizing that the compound contains an added bonus. Up to date, only the great apes have been found to exhibit optimal choices in this task, while macaques, dogs and pigeons fail to do so, generally showing an inability to process the compound item, and thus a bias toward the single item (Sánchez-Amaro et al., 2016). In contrast, in the *juice blending task*, the subjects make their choices only after witnessing the visual transformation of two of the three ingredients that are initially presented on the table, thus making a choice between a familiar ingredient and a novel mix, rather than choosing between an ingredient vs. a pair of ingredients presented side-by-side. Optimal performance in the *juice blending task* thus requires that the subjects understand that the visual transformation of the ingredients also entails a transformed taste–and thus hedonic–outcome. In turn, this raises demands for mental construction, and a kind of hedonic valuation that is best approximated by value and taste averaging (rather than summation), as single ingredients are up- or downgraded when mixed with other ingredients.

Whether combinatorial memory integration is a type of imagination which is within the apes’ cognitive repertoire is currently debated (for reviews see e.g., Whiten and Suddendorf, 2007; Abraham, 2016; Wechsler, 2019). Evidence suggests nevertheless that memory integration into novel situations is present in apes in a number of contexts, including their foraging decisions (as reviewed in the Introduction) or innovative tool uses that result from combining familiar behavioral sequences in novel ways (Davis et al., 2016; Vale et al., 2017). Other studies show that apes may exhibit even more complex forms of mental construction, which are indicative of rudimentary recursion–or the ability to hierarchically embed representations within representations. For example, they show an awareness of how others monitor their own attentional states (Hall et al., 2016) and a sensitivity to others’ false beliefs (e.g., Krupenye et al., 2016), they use pantomimes with compositional structure (as reviewed by Russon and Andrews, 2011) or map relations between relations (Hribar et al., 2011 and references therein).

Turning to relevant neural mechanisms, as already reviewed in the Introduction, the default mode network exhibits considerable anatomical and functional similarities in humans and chimpanzees (Rilling et al., 2007; Barks et al., 2015). Moreover, in the primate lineage, the size of a key structure implicated in mental construction–the vmPFC–co-varies with foraging complexity (Louail et al., 2019). This is consistent with accumulating evidence that a number of additional features of the prefrontal and medial cortex, which are implicated in a broad range of higher-order cognitive processes, are shared by humans with the other great apes, but less so with monkeys and lesser apes (e.g., Smaers et al., 2017; Amiez et al., 2019). This extends beyond measurements that target the size of relevant neural structures, to also include patterns of cortical serotonergic, dopaminergic and cholinergic innervation. Comparative analyses suggest a significant reorganization of such patterns in humans and chimpanzees as opposed to e.g., macaque monkeys, which in turn is consistent with greater cortical plasticity, behavioral flexibility, and memory function in Hominid species (Raghanti et al., 2008a, b, c). These differences notwithstanding, evidence from single cell recording studies with macaques shows that even primate species that are found to have less sophisticated cognitive abilities than chimpanzees exhibit neural structures able to implement an episodic system (for an extensive review, see Rolls and Wirth, 2018).

Before concluding, it is important to acknowledge the theoretical and methodological limitations that hinder an unequivocal interpretation concerning the cognitive mechanisms underlying the successful performance of chimpanzees in the *juice blending* task. With respect to theoretical limitations, additional work with human participants should clarify which novel situations raise requirements for AF and mental construction, which forms of hedonic predictions rely on episodic resources, what are the potential divergences between the neural mechanisms underlying explicit versus implicit mental construction, or if certain tasks described as involving AF (e.g., making predictions about novel food combination) could not be explained by other cognitive operations. For example, in a recent computational modeling study, the capability to make hedonic predictions about food combinations was described as a form of probabilistic reasoning, whereby the valuation of compounds depends on both ingredient values and the rules for combining them (Gershman et al., 2017). It is worth noting that the authors of this study too invoke AF as the cognitive mechanism involved in making hedonic predictions about food compounds. The implication of combinatory rules derived from extensive experience with compound valuation may appear inconsistent with AF being defined as an ability based on episodic construction. Yet, vast experience with compounds does not seem to be problematic to AF studies. Indeed, neuroimaging evidence shows that humans continue to resort to episodic construction when presented with novel food combinations (Barron et al., 2013), even though they most likely have accumulated a vast experience with compound foods. Most likely, generic experience with food compounding provides the semantic scaffolding necessary to all episodic construction in situations that require the evaluation of never-before experienced food compounds. Such an interplay between episodic and semantic resources is not specific to food blending tasks, but characterizes generally episodic constructive processes (Irish and Piguet, 2013; Szpunar et al., 2014).

In the same vein, it is conceivable that the chimpanzees in this study had some experience with compounds–e.g., when a piece of onion is consumed after a piece of tomato there is arguably some taste mixing. With repeated experience of this kind of mixing, it is further conceivable that the chimpanzees had formed some combinatorial rules for taste mixing concerning staple items in their diet, which, as a reminder, consists of vegetables and enriched pellets. This experience, however, should not impact the requirements for AF in our study, considering that the chimpanzees were presented with juice blends that were novel.

Since the phenomenological correlates of AF are understudied in humans, and, currently, inaccessible to researchers in apes, it is reasonable to describe the apes’ successful performance in the juice blending task as indicative of an AF-like ability (rather than human-like AF). This parallels the terminology in the comparative study of episodic memory, whereby the term episodic-like memory is used to refer to evidence that non-human animals integrate e.g., what-when-where information (e.g., Clayton and Dickinson, 1998).

The methodological limitations of the task derive from the small sample, and the fact that the version of the task employed here constitutes–in several ways–a minimal test of AF. Since our aim was to track the evolutionary origins of AF, in this task we focused on the core aspect of AF, i.e., on the ability to predict the hedonic outcome of novel situations given separate experience with elements of such novel situations. The task presented in this study was thus restricted to operationalizing this aspect, by capitalizing on culinary examples of AF, whereby humans are said to be able to predict the hedonic outcome of food combinations based on previous experience with ingredient foods, but without having directly experienced the combination (Wilson and Gilbert, 2003; Barron et al., 2013). As such, the task did not include any temporal displacement, as subjects were confronted with decisions concerning impending choices rather than having to project a more distant future. Although AF is described as being by default atemporal, AF is also regarded as a mechanism involved in certain forms of planning. The introduction of temporal displacement would thus link AF with planning. Future studies should thus include additional task manipulations, in order to reveal the extent and complexity of AF in apes. Understanding the ways in which AF in great apes–as compared to humans–is constrained by limitations in the processes that sub-serve it, will shed further light onto the evolutionary roots of this ability. Additional research with non-human species can generate new questions about AF mechanisms and thus stimulate additional theoretical and empirical research on AF in humans.

Currently, potential evidence of an AF-like ability in apes is limited to four datasets collected from three individuals–a Sumatran orangutan (Sauciuc et al., 2016) and two chimpanzees (present study). However, given the feeding ecology of the great apes (as described in the Introduction), and the potential adaptive value of AF in such a context, we expect rudimentary AF to be a trait generally expressed in these species. Indeed, neurobiological data suggest that, in the primate lineage, foraging complexity exerted selective pressures on a key brain structure known to support AF in humans, thus potentially leading to the emergence of AF in the last common ancestor of the great apes. In turn, this could have provided Hominids with the adaptive advantage to quickly evaluate mixed food patches using mental construction and hedonic simulation.

## Data Availability Statement

All datasets presented in this study are included in the Supplementary Material 1.

## Ethics Statement

Ethical review and approval was not required for the study on human participants in accordance with the local legislation and institutional requirements. The patients/participants provided their written informed consent to participate in this study. The animal study was reviewed and approved by Regional Ethical Review Board at Uppsala District Court (permit C110/15).

## Author Contributions

Both authors contributed equally to study conception, experimental design, and data collection. G-AS analyzed the data and drafted the manuscript. TP critically revised the manuscript.

## Conflict of Interest

The authors declare that the research was conducted in the absence of any commercial or financial relationships that could be construed as a potential conflict of interest.

## Footnotes

1 As used in this study, the notion of *simulation* refers to a basic computational brain mechanism that supports a broad range of perceptual and imaginative cognitive processes (e.g., Barsalou, 2009). A simulation is thus the hierarchical pattern of activation that is associated with a given experience. As such, seeing or thinking of a juice, implicates sensory and reward areas in the brain that process features such as color, texture, taste, and/or affective value, which are integrated (and coded for subsequent use) by conjunctive neurons in higher, cross-modal association areas. In the same vein, *mental construction* captures the parallel activation of simulations associated with distinct familiar experiences (e.g., pineapple juice and lemon juice), which engenders plasticity (new synapses) between the simultaneously active neural assemblies, thus supporting the construction of novel experiences (e.g., Barron et al., 2013).
