# Supplementary material for: Chimpanzees Predict the Hedonic Outcome of Novel Taste Combinations: The Evolutionary Origins of Affective Forecasting
Source: Front Psychol. 2020 Oct 6;11:549193. doi: 10.3389/fpsyg.2020.549193 (PMC7646213; doi:10.3389/fpsyg.2020.549193)
Supplement: Supplementary file 2 [file Table_1.docx]

**Supplementary Material 1 - Datasets**

Table 1: Overview of choice-derived preference scores and predicted preference score for the chimpanzees. Choice-derived preferences are expressed as percentages representing the number of times an item (ingredient or mix) was chosen across all occasions in which it was encountered.

| Maria Magdalena  Set 1 | Choice  derived  preferences | Predicted preference ranking | Maria  Magdalena  Set 2 | Choice  derived  preferences | Predicted preference ranking | Tjobbe | Choice  derived  preferences | Predicted preference ranking |
| --- | --- | --- | --- | --- | --- | --- | --- | --- |
| Apple | 17% | 2.00 | Cherry | 75% | 4.00 | Grapefruit | 42% | 2.00 |
| Apple & cherry | 50% | 3.00 | Cherry & lime | 75% | 2.50 | Grapefruit & lemon | 13% | 1.50 |
| Apple & lemon | 50% | 1.50 | Grapefruit | 33% | 2.00 | Grapefruit & pineapple | 50% | 2.50 |
| Apple & orange | 50% | 2.50 | Cherry & grapefruit | 88% | 3.00 | Lemon | 8% | 1.00 |
| Cherry | 83% | 4.00 | Grapefruit & pineapple | 63% | 2.50 | Lemon & pineapple | 38% | 2.00 |
| Cherry & lemon | 63% | 2.50 | Lime | 8% | 1.00 | Pineapple | 83% | 3.00 |
| Lemon & orange | 75% | 2.00 | Lime & grapefruit | 50% | 1.50 | Pomegranate & grapefruit | 63% | 3.00 |
| Orange | 58% | 3.00 | Lime & pineapple | 13% | 2.00 | Pomegranate | 92% | 4.00 |
| Orange & cherry | 75% | 3.50 | Pineapple | 25% | 3.00 | Pomegranate & lemon | 50% | 2.50 |
| Lemon | 0% | 1.00 | Pineapple & cherry | 88% | 3.50 | Pomegranate & pineapple | 75% | 3.50 |

Datasets 1-14: Test trials for the chimpanzees tested for the purposes of this study, as well as for the human participants and Sumatran orangutan tested for the purposes of Sauciuc et al. (2016). Each dataset has been annotated to include the ingredient ranking and the predicted ranking for the respective individual. Key trials (as defined and analyzed in the Results section) are highlighted in yellow. Tie-trials (as defined and analyzed in the Results section) are highlighted in orange. To ease data scrutiny, the trials are not presented in randomized order, but in a standardized format, with the familiar ingredient being always presented on the left and the novel mix on the right. Moreover, within a block of trials, the trials are presented in the alphabetical order of the ingredients.

| **P1 (HUMAN)** | block no. | INGREDIENT | MIX | CHOICE | MATCH TO PREDICTION |
| --- | --- | --- | --- | --- | --- |
| ingredient ranking: R > C > L > V | 1 | C | R-V | C | YES |
| predicted ranking: R > CR > C = LR > CL = VR > CV = L > VL > V | 1 | C | C-V | C | YES |
| C = cherry | 1 | C | C-L | CL | **NO** |
| L = diluted lemon | 1 | C | C-R | C-R | YES |
| R = rhubarb | 1 | C | L-R | C | YES |
| V = diluted vinegar | 1 | C | L-V | C | YES |
| yellow highlight = key trials | 1 | L | C-L | C-L | YES |
| orange highlight = tie trials | 1 | L | C-R | C-R | YES |
|  | 1 | L | C-V | C-V | YES |
|  | 1 | L | L-V | L | YES |
|  | 1 | L | L-R | L-R | YES |
|  | 1 | L | R-V | R-V | YES |
|  | 1 | R | C-L | C-L | **NO** |
|  | 1 | R | L-R | R | YES |
|  | 1 | R | L-V | R | YES |
|  | 1 | R | C-R | C-R | **NO** |
|  | 1 | R | C-V | R | YES |
|  | 1 | R | R-V | R-V | **NO** |
|  | 1 | V | C-L | C-L | YES |
|  | 1 | V | C-R | C-R | YES |
|  | 1 | V | L-R | L-R | YES |
|  | 1 | V | C-V | C-V | YES |
|  | 1 | V | R-V | R-V | YES |
|  | 1 | V | L-V | L-V | YES |
|  | 2 | C | C-R | C-R | YES |
|  | 2 | C | L-R | C | YES |
|  | 2 | C | R-V | C | YES |
|  | 2 | C | C-L | C | YES |
|  | 2 | C | C-V | C | YES |
|  | 2 | C | L-V | C | YES |
|  | 2 | L | C-R | C-R | YES |
|  | 2 | L | C-V | L | YES |
|  | 2 | L | C-L | C-L | YES |
|  | 2 | *L* | *L-R* | *X* | *MISS* |
|  | 2 | L | L-V | L | YES |
|  | 2 | L | R-V | L | **NO** |
|  | 2 | R | C-L | R | YES |
|  | 2 | R | C-R | C-R | **NO** |
|  | 2 | R | L-R | R | YES |
|  | 2 | R | C-V | R | YES |
|  | 2 | R | R-V | R | YES |
|  | 2 | R | L-V | R | YES |
|  | 2 | V | C-V | C-V | YES |
|  | 2 | V | L-R | L-R | YES |
|  | 2 | V | L-V | L-V | YES |
|  | 2 | V | C-L | C-L | YES |
|  | 2 | V | C-R | C-R | YES |
|  | 2 | V | R-V | RV | YES |
|  |  |  |  |  |  |
| **P2 (HUMAN)** | block no. | INGREDIENT | MIX | CHOICE | MATCH TO PREDICTION |
| ingredient ranking: R > C > L > V | 1 | C | R-V | C | YES |
| predictive ranking: R > CR > C = LR > CL = VR > CV = L > VL > V | 1 | C | C-L | C-L | **NO** |
| C = cherry | 1 | C | C-V | C | YES |
| L = diluted lemon | 1 | C | L-V | L-V | **NO** |
| R = rhubarb | 1 | C | C-R | C-R | YES |
| V = diluted vinegar | 1 | C | L-R | C | YES |
| yellow highlight = key trials | 1 | L | C-L | L | **NO** |
| orange highlight = tie trials | 1 | L | C-R | C-R | YES |
|  | 1 | L | R-V | L | **NO** |
|  | 1 | L | L-V | L | YES |
|  | 1 | L | L-R | L-R | YES |
|  | 1 | L | C-V | L | YES |
|  | 1 | R | C-L | R | YES |
|  | 1 | R | L-R | R | YES |
|  | 1 | R | C-R | R | YES |
|  | 1 | R | C-V | R | YES |
|  | 1 | R | L-V | R | YES |
|  | 1 | R | R-V | R | YES |
|  | 1 | V | C-L | C-L | YES |
|  | 1 | V | L-R | L-R | YES |
|  | 1 | V | C-V | C-V | YES |
|  | 1 | V | R-V | R-V | YES |
|  | 1 | V | L-V | L-V | YES |
|  | 1 | V | C-R | C-R | YES |
|  | 2 | C | L-R | C | YES |
|  | 2 | C | C-R | C-R | YES |
|  | 2 | C | R-V | VR | **NO** |
|  | 2 | C | C-L | C | YES |
|  | 2 | C | C-V | C | YES |
|  | 2 | C | L-V | C | YES |
|  | 2 | L | C-R | C-R | YES |
|  | 2 | L | C-V | L | YES |
|  | 2 | L | C-L | C-L | YES |
|  | 2 | L | L-R | L-R | YES |
|  | 2 | L | L-V | L | YES |
|  | 2 | L | R-V | L | **NO** |
|  | 2 | R | C-L | R | YES |
|  | 2 | R | C-R | R | YES |
|  | 2 | R | L-R | R | YES |
|  | 2 | R | C-V | R | YES |
|  | 2 | R | R-V | R | YES |
|  | 2 | R | L-V | R | YES |
|  | 2 | V | C-V | V | **NO** |
|  | 2 | V | L-R | LR | YES |
|  | 2 | V | C-R | C-R | YES |
|  | 2 | V | R-V | R-V | YES |
|  | 2 | V | C-L | C-L | YES |
|  | 2 | V | L-V | L-V | YES |
|  |  |  |  |  |  |
| **P3 (HUMAN)** | block no. | INGREDIENT | MIX | CHOICE | MATCH TO PREDICTION |
| ingredient ranking: R > C > L > V | 1 | C | R-V | C | YES |
| predictive ranking: R > CR > C = LR > CL = VR > CV = L > VL > V | 1 | C | C-L | C | YES |
| C = cherry | 1 | C | C-V | C | YES |
| L = diluted lemon | 1 | C | C-R | C-R | YES |
| R = rhubarb | 1 | C | L-R | C | YES |
| V = diluted vinegar | 1 | C | L-V | C | YES |
| yellow highlight = key trials | 1 | L | C-L | L | **NO** |
| orange highlight = tie trials | 1 | L | C-R | C-R | YES |
|  | 1 | L | C-V | L | YES |
|  | 1 | L | R-V | L | YES |
|  | 1 | L | L-V | L | YES |
|  | 1 | L | L-R | L-R | YES |
|  | 1 | R | C-L | R | YES |
|  | 1 | R | L-R | R | YES |
|  | 1 | R | L-V | R | YES |
|  | 1 | R | C-R | R | YES |
|  | 1 | R | C-V | R | YES |
|  | 1 | R | R-V | R | YES |
|  | 1 | V | C-L | C-L | YES |
|  | 1 | V | C-R | C-R | YES |
|  | 1 | V | L-R | L-R | YES |
|  | 1 | V | L-V | L-V | YES |
|  | 1 | V | C-V | C-V | YES |
|  | 1 | V | R-V | R-V | YES |
|  | 2 | C | L-R | C | YES |
|  | 2 | C | C-R | C | **NO** |
|  | 2 | C | R-V | C | YES |
|  | 2 | C | L-V | C | YES |
|  | 2 | C | C-V | C | YES |
|  | 2 | C | C-L | C-L | **NO** |
|  | 2 | L | C-L | C-L | YES |
|  | 2 | L | C-V | L | YES |
|  | 2 | L | C-R | L | **NO** |
|  | 2 | L | R-V | L | **NO** |
|  | 2 | L | L-V | L | YES |
|  | 2 | L | L-R | L-R | YES |
|  | 2 | R | C-L | C-L | **NO** |
|  | 2 | R | C-R | C-R | **NO** |
|  | 2 | R | L-R | R | YES |
|  | 2 | R | C-V | R | YES |
|  | 2 | R | L-V | R | YES |
|  | 2 | R | R-V | R | YES |
|  | 2 | V | C-L | C-L | YES |
|  | 2 | V | C-R | C-R | YES |
|  | 2 | V | C-V | C-V | YES |
|  | 2 | V | L-R | L-R | YES |
|  | 2 | V | L-V | L-V | YES |
|  | 2 | V | R-V | R-V | YES |
|  |  |  |  |  |  |
| **P4 (HUMAN)** | block no. | INGREDIENT | MIX | CHOICE | MATCH TO PREDICTION |
| ingredient ranking: R > C > L > V | 1 | C | R-V | C | YES |
| predictive ranking: R > CR > C = LR > CL = VR > L = CV > VL > V | 1 | C | C-V | C | YES |
| C = cherry | 1 | C | C-L | C-L | **NO** |
| L = diluted lemon | 1 | C | C-R | C-R | YES |
| R = rhubarb | 1 | C | L-R | C | YES |
| V = diluted vinegar | 1 | C | L-V | C | YES |
| yellow highlight = key trials | 1 | L | C-L | C-L | YES |
| orange highlight = tie trials | 1 | L | C-R | L | **NO** |
|  | 1 | L | C-V | L | YES |
|  | 1 | L | L-V | L | YES |
|  | 1 | L | L-R | L-R | YES |
|  | 1 | L | R-V | R-V | YES |
|  | 1 | R | C-L | R | YES |
|  | 1 | R | C-R | C-R | **NO** |
|  | 1 | R | C-V | C-V | **NO** |
|  | 1 | R | L-R | R | YES |
|  | 1 | R | L-V | R | YES |
|  | 1 | R | R-V | R | YES |
|  | 1 | V | C-L | C-L | YES |
|  | 1 | V | C-R | C-R | YES |
|  | 1 | V | L-R | LR | YES |
|  | 1 | V | L-V | L-V | YES |
|  | 1 | V | C-V | C-V | YES |
|  | 1 | V | R-V | R-V | YES |
|  | 2 | C | L-R | C | YES |
|  | 2 | C | C-R | C-R | YES |
|  | 2 | C | R-V | R-V | **NO** |
|  | 2 | C | C-L | C | YES |
|  | 2 | C | L-V | C | YES |
|  | 2 | C | C-V | C | YES |
|  | 2 | L | C-V | L | YES |
|  | 2 | L | C-L | C-L | YES |
|  | 2 | L | L-V | L | YES |
|  | 2 | L | L-R | L-R | YES |
|  | 2 | L | R-V | L | **NO** |
|  | 2 | L | C-R | C-R | YES |
|  | 2 | R | C-R | C-R | **NO** |
|  | 2 | R | C-V | R | YES |
|  | 2 | R | R-V | R-V | **NO** |
|  | 2 | R | L-V | R | YES |
|  | 2 | R | C-L | R | YES |
|  | 2 | R | L-R | L-R | **NO** |
|  | 2 | V | C-V | C-V | YES |
|  | 2 | V | C-L | C-L | YES |
|  | 2 | V | C-R | C-R | YES |
|  | 2 | V | R-V | R-V | YES |
|  | 2 | V | L-V | L-V | YES |
|  | 2 | V | L-R | L-R | YES |
|  |  |  |  |  |  |
| **P5 (HUMAN)** | block no. | INGREDIENT | MIX | CHOICE | MATCH TO PREDICTION |
| ingredient ranking: L > R > C > V | 1 | C | R-V | C | YES |
| predictive ranking: L > LR > R = CL > CR = VL > C = VR > CV > V | 1 | C | C-L | C-L | YES |
| C = cherry | 1 | C | C-V | C | YES |
| L = diluted lemon | 1 | C | C-R | C-R | YES |
| R = rhubarb | 1 | C | L-R | L-R | YES |
| V = diluted vinegar | 1 | C | L-V | C | YES |
| yellow highlight = key trials | 1 | L | R-V | L | YES |
| orange highlight = tie trials | 1 | L | L-V | L | YES |
|  | 1 | L | L-R | L-R | **NO** |
|  | 1 | L | C-L | L | YES |
|  | 1 | L | C-R | L | YES |
|  | 1 | L | C-V | L | YES |
|  | 1 | R | C-L | C-L | YES |
|  | 1 | R | L-R | L-R | YES |
|  | 1 | R | C-R | C-R | **NO** |
|  | 1 | R | C-V | R | YES |
|  | 1 | R | L-V | R | YES |
|  | 1 | R | R-V | R | YES |
|  | 1 | V | C-L | C-L | YES |
|  | 1 | V | C-V | C-V | YES |
|  | 1 | V | R-V | R-V | YES |
|  | 1 | V | V | L-V | YES |
|  | 1 | V | C-R | C-R | YES |
|  | 1 | V | L-R | L-R | YES |
|  | 2 | C | L-R | L-R | YES |
|  | 2 | C | C-R | C-R | YES |
|  | 2 | C | R-V | C | YES |
|  | 2 | C | C-V | C | YES |
|  | 2 | C | L-V | C | **NO** |
|  | 2 | C | C-L | C-L | YES |
|  | 2 | L | C-R | L | YES |
|  | 2 | L | C-V | L | YES |
|  | 2 | L | C-L | C-L | **NO** |
|  | 2 | L | L-R | L | YES |
|  | 2 | L | L-V | L | YES |
|  | 2 | L | R-V | L | YES |
|  | 2 | R | C-L | C-L | YES |
|  | 2 | R | C-R | C-R | **NO** |
|  | 2 | R | L-R | L-R | YES |
|  | 2 | R | C-V | R | YES |
|  | 2 | R | R-V | R | YES |
|  | 2 | R | L-V | R | YES |
|  | 2 | V | C-V | C-V | YES |
|  | 2 | V | C-R | C-R | YES |
|  | 2 | V | R-V | R-V | YES |
|  | 2 | V | C-L | C-L | YES |
|  | 2 | V | L-V | L-V | YES |
|  | 2 | V | L-R | L-R | YES |
|  |  |  |  |  |  |
| **P6 (HUMAN)** | block no. | INGREDIENT | MIX | CHOICE | MATCH TO PREDICTION |
| ingredient ranking: R > C > V > L | 1 | C | R-V | R-V | YES |
| predictive ranking: R > CR > C = VR > CV = LR > CL = V > VL > L | 1 | C | C-L | C | YES |
| C = cherry | 1 | C | C-V | C | YES |
| L = diluted lemon | 1 | C | C-R | C | **NO** |
| R = rhubarb | 1 | C | L-R | C | YES |
| V = diluted vinegar | 1 | C | L-V | L-V | **NO** |
| yellow highlight = key trials | 1 | L | C-L | L | **NO** |
| orange highlight = tie trials | 1 | L | C-R | L | **NO** |
|  | 1 | L | C-V | L | **NO** |
|  | 1 | L | R-V | R-V | YES |
|  | 1 | L | L-V | L-V | YES |
|  | 1 | L | L-R | L-R | YES |
|  | 1 | R | C-L | R | YES |
|  | 1 | R | L-R | R | YES |
|  | 1 | R | C-R | C-R | YES |
|  | 1 | R | C-V | R | YES |
|  | 1 | R | L-V | R | YES |
|  | 1 | R | R-V | R | YES |
|  | 1 | V | C-L | C-L | YES |
|  | 1 | V | C-R | C-R | YES |
|  | 1 | V | L-R | L-R | YES |
|  | 1 | V | C-V | CV | YES |
|  | 1 | V | R-V | V | **NO** |
|  | 1 | V | L-V | V | YES |
|  | 2 | C | L-R | C | YES |
|  | 2 | C | C-R | C | **NO** |
|  | 2 | C | R-V | C | TIE/YES |
|  | 2 | C | C-L | C | YES |
|  | 2 | C | C-V | C | YES |
|  | 2 | C | L-V | C | YES |
|  | 2 | L | C-R | C-R | YES |
|  | 2 | L | C-V | L | **NO** |
|  | 2 | L | C-L | C-L | YES |
|  | 2 | L | L-R | L-R | YES |
|  | 2 | L | L-V | L-V | YES |
|  | 2 | L | R-V | R-V | YES |
|  | 2 | R | C-L | R | YES |
|  | 2 | R | C-R | R | YES |
|  | 2 | R | L-R | R | YES |
|  | 2 | R | C-V | C-V | **NO** |
|  | 2 | R | R-V | R | YES |
|  | 2 | R | L-V | R | YES |
|  | 2 | V | C-V | C-V | YES |
|  | 2 | V | L-R | L-R | YES |
|  | 2 | V | C-R | C-R | YES |
|  | 2 | V | R-V | R-V | YES |
|  | 2 | V | C-L | C-L | YES |
|  | 2 | V | L-V | V | YES |
|  |  |  |  |  |  |
| **P7 (HUMAN)** | block no. | INGREDIENT | MIX | CHOICE | MATCH TO PREDICTION |
| ingredient ranking: C > R > L > V | 1 | C | R-V | C | YES |
| predictive ranking: C > CR > R =CL > CV = LR > L = VR > VL > V | 1 | C | C-L | C | YES |
| C = cherry | 1 | C | C-V | C | YES |
| L = diluted lemon | 1 | C | C-R | C | YES |
| R = rhubarb | 1 | C | L-R | C | YES |
| V = diluted vinegar | 1 | C | L-V | C | YES |
| yellow highlight = key trials | 1 | L | C-L | C-L | YES |
| orange highlight = tie trials | 1 | L | C-R | C-R | YES |
|  | 1 | L | C-V | C-V | YES |
|  | 1 | L | R-V | L | YES |
|  | 1 | L | L-V | L | YES |
|  | 1 | L | L-R | L-R | YES |
|  | 1 | R | C-L | R | YES |
|  | 1 | R | L-R | R | YES |
|  | 1 | R | C-R | C-R | YES |
|  | 1 | R | C-V | R | YES |
|  | 1 | R | L-V | R | YES |
|  | 1 | R | R-V | R | YES |
|  | 1 | V | C-L | C-L | YES |
|  | 1 | V | C-R | C-R | YES |
|  | 1 | V | L-R | L-R | YES |
|  | 1 | V | C-V | C-V | YES |
|  | 1 | V | R-V | R-V | YES |
|  | 1 | V | L-V | L-V | YES |
|  | 2 | C | L-R | C | YES |
|  | 2 | C | C-R | C-R | **NO** |
|  | 2 | C | R-V | R-V | **NO** |
|  | 2 | C | C-L | C | YES |
|  | 2 | C | C-V | C | YES |
|  | 2 | C | L-V | C | YES |
|  | 2 | L | C-R | C-R | YES |
|  | 2 | L | C-V | L | **NO** |
|  | 2 | L | C-L | C-L | YES |
|  | 2 | L | L-R | L-R | YES |
|  | 2 | L | L-V | L | YES |
|  | 2 | L | R-V | L | YES |
|  | 2 | R | C-L | R | YES |
|  | 2 | R | C-R | C-R | YES |
|  | 2 | R | L-R | R | YES |
|  | 2 | R | C-V | R | YES |
|  | 2 | R | R-V | R | YES |
|  | 2 | R | L-V | R | YES |
|  | 2 | V | C-V | C-V | YES |
|  | 2 | V | L-R | L-R | YES |
|  | 2 | V | C-R | C-R | YES |
|  | 2 | V | R-V | R-V | YES |
|  | 2 | V | C-L | C-L | YES |
|  | 2 | V | L-V | L-V | YES |
|  |  |  |  |  |  |
| **P8 (HUMAN)** | block no. | INGREDIENT | MIX | CHOICE | MATCH TO PREDICTION |
| ingredient ranking: R > C > V > L | 1 | C | R-V | R-V | YES |
| predictive ranking: R > CR > C = VR > CV = LR > CL = V > VL > L | 1 | C | C-L | C | YES |
| C = cherry | 1 | C | C-V | C | YES |
| L = diluted lemon | 1 | C | C-R | C-R | YES |
| R = rhubarb | 1 | C | L-R | C | YES |
| V = diluted vinegar | 1 | C | L-V | L-V | **NO** |
| yellow highlight = key trials | 1 | L | C-L | C-L | YES |
| orange highlight = tie trials | 1 | L | C-R | C-R | YES |
|  | 1 | L | C-V | C-V | YES |
|  | 1 | L | R-V | R-V | YES |
|  | 1 | L | L-V | L-V | YES |
|  | 1 | L | L-R | L-R | YES |
|  | 1 | R | C-L | R | YES |
|  | 1 | R | L-R | R | YES |
|  | 1 | R | C-R | C-R | **NO** |
|  | 1 | R | C-V | R | YES |
|  | 1 | R | L-V | R | YES |
|  | 1 | R | R-V | R | YES |
|  | 1 | V | C-L | C-L | YES |
|  | 1 | V | C-R | C-R | YES |
|  | 1 | V | L-R | V | **NO** |
|  | 1 | V | C-V | C-V | YES |
|  | 1 | V | R-V | R-V | YES |
|  | 1 | V | L-V | V | YES |
|  | 2 | C | L-R | C | YES |
|  | 2 | C | C-R | C-R | YES |
|  | 2 | C | R-V | C | YES |
|  | 2 | C | C-L | C | YES |
|  | 2 | C | C-V | C | YES |
|  | 2 | C | L-V | C | YES |
|  | 2 | L | C-R | C-R | YES |
|  | 2 | L | C-V | C-V | YES |
|  | 2 | L | C-L | C-L | YES |
|  | 2 | L | L-R | L-R | YES |
|  | 2 | L | L-V | L-V | YES |
|  | 2 | L | R-V | R-V | YES |
|  | 2 | R | C-L | R | YES |
|  | 2 | R | C-R | C-R | **NO** |
|  | 2 | R | L-R | R | YES |
|  | 2 | R | C-V | R | YES |
|  | 2 | R | R-V | R | YES |
|  | 2 | R | L-V | R | YES |
|  | 2 | V | C-V | C-V | YES |
|  | 2 | V | L-R | V | **NO** |
|  | 2 | V | C-R | C-R | YES |
|  | 2 | V | R-V | R-V | YES |
|  | 2 | V | C-L | V | YES |
|  | 2 | V | L-V | V | YES |
|  |  |  |  |  |  |
| **P9 (HUMAN)** | block no. | INGREDIENT | MIX | CHOICE | MATCH TO PREDICTION |
| ingredient ranking: L > R > C > V | 1 | C | L-V | C | **NO** |
| predictive ranking: L > LR > CL = R > CR = VL > C = VR > CV > V | 1 | C | R-V | C | YES |
| C = cherry | 1 | C | C-L | C | **NO** |
| L = diluted lemon | 1 | C | C-V | C | YES |
| R = rhubarb | 1 | C | C-R | C-R | YES |
| V = diluted vinegar | 1 | C | L-R | L-R | YES |
| yellow highlight = key trials | 1 | L | C-L | L | YES |
| orange highlight = tie trials | 1 | L | C-R | L | YES |
|  | 1 | L | C-V | L | YES |
|  | 1 | L | R-V | L | YES |
|  | 1 | L | L-V | L | YES |
|  | 1 | L | L-R | L-R | **NO** |
|  | 1 | R | C-L | C-L | YES |
|  | 1 | R | L-R | L-R | YES |
|  | 1 | R | C-R | R | YES |
|  | 1 | R | C-V | R | YES |
|  | 1 | R | L-V | R | YES |
|  | 1 | R | R-V | R | YES |
|  | 1 | V | C-L | C-L | YES |
|  | 1 | V | C-R | C-R | YES |
|  | 1 | V | L-R | L-R | YES |
|  | 1 | V | C-V | C-V | YES |
|  | 1 | V | R-V | R-V | YES |
|  | 1 | V | V-L | V-L | YES |
|  | 2 | C | L-R | L-R | YES |
|  | 2 | C | C-R | C-R | YES |
|  | 2 | C | R-V | C | YES |
|  | 2 | C | C-L | C-L | YES |
|  | 2 | C | C-V | C | YES |
|  | 2 | C | L-V | C | **NO** |
|  | 2 | L | C-R | L | YES |
|  | 2 | L | C-V | L | YES |
|  | 2 | L | C-L | C-L | **NO** |
|  | 2 | L | L-R | L-R | **NO** |
|  | 2 | L | L-V | L | YES |
|  | 2 | L | R-V | L | YES |
|  | 2 | R | C-L | C-L | YES |
|  | 2 | R | C-R | R | YES |
|  | 2 | R | L-R | L-R | YES |
|  | 2 | R | C-V | R | YES |
|  | 2 | R | R-V | R | YES |
|  | 2 | R | L-V | R | YES |
|  | 2 | V | C-V | C-V | YES |
|  | 2 | V | L-R | L-R | YES |
|  | 2 | V | C-R | C-R | YES |
|  | 2 | V | R-V | R-V | YES |
|  | 2 | V | C-L | C-L | YES |
|  | 2 | V | L-V | L-V | YES |
|  |  |  |  |  |  |
| **P10 (HUMAN)** | block no. | INGREDIENT | MIX | CHOICE | MATCH TO PREDICTION |
| ingredient ranking: L > R > C > V | 1 | C | R-V | C | YES |
| predictive ranking: L > LR > R = CL > CR = VL > C = VR > CV > V | 1 | C | C-L | C-L | YES |
| C = cherry | 1 | C | C-V | C-V | **NO** |
| L = diluted lemon | 1 | C | C-R | C-R | YES |
| R = rhubarb | 1 | C | L-R | L-R | YES |
| V = diluted vinegar | 1 | C | L-V | C | **NO** |
| yellow highlight = key trials | 1 | L | C-L | C-L | **NO** |
| orange highlight = tie trials | 1 | L | C-R | L | YES |
|  | 1 | L | C-V | L | YES |
|  | 1 | L | R-V | L | YES |
|  | 1 | L | L-V | L-V | **NO** |
|  | 1 | L | L-R | L-R | **NO** |
|  | 1 | R | C-L | C-L | YES |
|  | 1 | R | L-R | L-R | YES |
|  | 1 | R | C-R | C-R | **NO** |
|  | 1 | R | C-V | R | YES |
|  | 1 | R | L-V | R | YES |
|  | 1 | R | R-V | R-V | **NO** |
|  | 1 | V | C-L | C-L | YES |
|  | 1 | V | C-R | C-R | YES |
|  | 1 | V | L-R | L-R | YES |
|  | 1 | V | C-V | C-V | YES |
|  | 1 | V | R-V | R-V | YES |
|  | 1 | V | L-V | L-V | YES |
|  | 2 | C | L-R | L-R | YES |
|  | 2 | C | C-R | C-R | YES |
|  | 2 | C | R-V | C | YES |
|  | 2 | C | C-L | C-L | YES |
|  | 2 | C | C-V | C | YES |
|  | 2 | C | L-V | C | **NO** |
|  | 2 | L | C-R | C-R | **NO** |
|  | 2 | L | C-V | L | YES |
|  | 2 | L | C-L | C-L | **NO** |
|  | 2 | L | L-R | L-R | **NO** |
|  | 2 | L | L-V | L | YES |
|  | 2 | L | R-V | L | YES |
|  | 2 | R | C-L | C-L | YES |
|  | 2 | R | C-R | R | YES |
|  | 2 | R | L-R | L-R | YES |
|  | 2 | R | C-V | R | YES |
|  | 2 | R | R-V | R | YES |
|  | 2 | R | L-V | R | YES |
|  | 2 | V | C-V | C-V | YES |
|  | 2 | V | L-R | L-R | YES |
|  | 2 | V | C-R | C-R | YES |
|  | 2 | V | R-V | R-V | YES |
|  | 2 | V | C-L | C-L | YES |
|  | 2 | V | L-V | L-V | YES |

| **TJOBBE (CHIMPANZEE)** | block no. | INGREDIENT | MIX | CHOICE | MATCH TO PREDICTION |
| --- | --- | --- | --- | --- | --- |
| ingredient ranking: PO > P > G > L | 1 | G | PO-P | PO-P | YES |
| predicted ranking: PO > POP > P = POG > GP = POL > G = LP > LG > L | 1 | G | G-L | G | YES |
| PO = pomegranate | 1 | G | G-P | G | **NO** |
| P = pineapple | 1 | G | PO-G | PO-G | YES |
| G = grapefruit | 1 | G | PO-L | PO-L | YES |
| L = lemon | 1 | G | P-L | g | YES |
| yellow highlight = key trials | 1 | L | G-L | L | **NO** |
| orange highlight = tie trials | 1 | L | G-P | G-P | YES |
|  | 1 | L | L-P | L-P | YES |
|  | 1 | L | PO-G | PO-G | YES |
|  | 1 | L | PO-L | PO-L | YES |
|  | 1 | L | PO-P | PO-P | YES |
|  | 1 | P | G-L | P | YES |
|  | 1 | P | G-P | P | YES |
|  | 1 | P | L-P | P | YES |
|  | 1 | P | PO-G | PO-G | YES |
|  | 1 | P | PO-L | P | YES |
|  | 1 | P | PO-P | P | **NO** |
|  | 1 | PO | G-L | PO | YES |
|  | 1 | PO | G-P | PO | YES |
|  | 1 | PO | L-P | PO | YES |
|  | 1 | PO | PO-G | PO | YES |
|  | 1 | PO | PO-L | PO-L | **NO** |
|  | 1 | PO | PO-P | PO | YES |
|  | 2 | G | PO-P | PO-P | YES |
|  | 2 | G | G-L | G | YES |
|  | 2 | G | G-P | G-P | YES |
|  | 2 | G | PO-G | PO-G | YES |
|  | 2 | G | PO-L | PO-L | YES |
|  | 2 | G | P-L | P-L | YES |
|  | 2 | L | G-L | gl | YES |
|  | 2 | L | G-P | G-P | YES |
|  | 2 | L | L-P | lp | YES |
|  | 2 | L | PO-G | PO-G | YES |
|  | 2 | L | PO-L | PO-L | YES |
|  | 2 | L | PO-P | PO-P | YES |
|  | 2 | P | G-L | P | YES |
|  | 2 | P | G-P | G-P | **NO** |
|  | 2 | P | L-P | P | YES |
|  | 2 | P | PO-G | P | YES |
|  | 2 | P | PO-L | P | YES |
|  | 2 | P | PO-P | P | **NO** |
|  | 2 | PO | G-L | PO | YES |
|  | 2 | PO | G-P | PO | YES |
|  | 2 | PO | L-P | PO | YES |
|  | 2 | PO | PO-G | PO | YES |
|  | 2 | PO | PO-L | PO | YES |
|  | 2 | PO | PO-P | PO | YES |
|  |  |  |  |  |  |
| **MARIA MAGDALENA SET 1 (CHIMPANZEE)** | block no. | INGREDIENT | MIX | CHOICE | MATCH TO PREDICTION |
| ingredient ranking: C > O > A > L | 1 | A | L-O | L-O | YES |
| predicted ranking: C > OC > AC = O > AO = CL > A = LO > AL > L | 1 | A | C-L | C-L | YES |
| C = cherry | 1 | A | C-O | C-O | YES |
| O = orange | 1 | A | A-L | A-L | **NO** |
| A = apple | 1 | A | C-A | C-A | YES |
| L = lemon | 1 | A | A-O | A-O | YES |
| yellow highlight = key trials | 1 | C | A-O | C | YES |
| orange highlight = tie trials | 1 | C | A-L | C | YES |
|  | 1 | C | L-O | L-O | **NO** |
|  | 1 | C | C-O | C | YES |
|  | 1 | C | A-C | C | YES |
|  | 1 | L | A-L | A-L | YES |
|  | 1 | L | A-O | A-O | YES |
|  | 1 | L | C-L | C-L | YES |
|  | 1 | L | C-O | C-O | YES |
|  | 1 | L | A-C | A-C | YES |
|  | 1 | L | C-L | C | YES |
|  | 1 | L | L-O | L-O | YES |
|  | 1 | O | A-C | O | YES |
|  | 1 | O | C-O | C-O | YES |
|  | 1 | O | L-O | O | YES |
|  | 1 | O | A-O | O | YES |
|  | 1 | O | C-L | C-L | **NO** |
|  | 1 | O | A-L | O | YES |
|  | 2 | A | L-O | L-O | YES |
|  | 2 | A | C-L | A | **NO** |
|  | 2 | A | C-O | o c | YES |
|  | 2 | A | A-L | A | YES |
|  | 2 | A | C-A | C-A | YES |
|  | 2 | A | A-O | A-O | YES |
|  | 2 | C | A-O | C | YES |
|  | 2 | C | A-L | C | YES |
|  | 2 | C | L-O | C | YES |
|  | 2 | C | C-O | C | YES |
|  | 2 | C | A-C | C | YES |
|  | 2 | L | A-L | A-L | YES |
|  | 2 | L | A-O | A-O | YES |
|  | 2 | L | C-L | C-L | YES |
|  | 2 | L | C-O | C-O | YES |
|  | 2 | L | A-C | A-C | YES |
|  | 2 | L | C-L | C-L | **NO** |
|  | 2 | L | L-O | L-O | YES |
|  | 2 | O | A-C | O | YES |
|  | 2 | O | C-O | C-O | YES |
|  | 2 | O | L-O | L-O | **NO** |
|  | 2 | O | A-O | O | YES |
|  | 2 | O | C-L | O | YES |
|  | 2 | O | A-L | A-L | **NO** |
|  |  |  |  |  |  |
| **MARIA MAGDALENA SET 2 (CHIMPANZEE)** | block no. | INGREDIENT | MIX | CHOICE | MATCH TO PREDICTION |
| ingredient ranking: C > P > G > L | 1 | C | C-L | C-L | **NO** |
| predicted ranking: C > CP > CG = P > CL = GP > G = LP > GL > L | 1 | C | G-P | G-P | **NO** |
| C = cherry | 1 | C | C-P | C-P | **NO** |
| G = grapefruit | 1 | C | C-G | C | YES |
| L = lime | 1 | C | G-L | C | YES |
| P = pineapple | 1 | C | L-P | C | YES |
| yellow highlight = key trials | 1 | G | C-G | C-G | YES |
| orange highlight = tie trials | 1 | G | L-P | G | YES |
|  | 1 | G | C-L | C-L | YES |
|  | 1 | G | C-P | C-P | YES |
|  | 1 | G | G-P | G-P | YES |
|  | 1 | G | G-L | G | YES |
|  | 1 | L | C-L | C-L | YES |
|  | 1 | L | C-G | C-G | YES |
|  | 1 | L | G-P | G-P | YES |
|  | 1 | L | C-P | C-P | YES |
|  | 1 | L | G-L | G-L | YES |
|  | 1 | L | L-P | L | **NO** |
|  | 1 | P | C-G | C-G | YES |
|  | 1 | P | G-P | P | YES |
|  | 1 | P | G-L | G-L | **NO** |
|  | 1 | P | C-P | C-P | YES |
|  | 1 | P | C-L | C-L | **NO** |
|  | 1 | P | L-P | P | YES |
|  | 2 | C | C-L | C | YES |
|  | 2 | C | G-P | C | YES |
|  | 2 | C | C-P | C | YES |
|  | 2 | C | C-G | C | YES |
|  | 2 | C | G-L | C | YES |
|  | 2 | C | L-P | C | YES |
|  | 2 | G | C-G | C-G | YES |
|  | 2 | G | L-P | G | YES |
|  | 2 | G | C-L | C-L | YES |
|  | 2 | G | C-P | C-P | YES |
|  | 2 | G | G-P | G | **NO** |
|  | 2 | G | G-L | G | YES |
|  | 2 | L | C-L | C-L | YES |
|  | 2 | L | C-G | C-G | YES |
|  | 2 | L | G-P | G-P | YES |
|  | 2 | L | C-P | C-P | YES |
|  | 2 | L | G-L | G-L | YES |
|  | 2 | L | L-P | L-P | YES |
|  | 2 | P | C-G | C-G | YES |
|  | 2 | P | G-P | G-P | **NO** |
|  | 2 | P | G-L | G-L | **NO** |
|  | 2 | P | C-P | C-P | YES |
|  | 2 | P | C-L | C-L | **NO** |
|  | 2 | P | L-P | P | YES |

| **NAONG (SUMATRAN ORANGUTAN)** | block no. | INGREDIENT | MIX | CHOICE | MATCH TO PREDICTION |
| --- | --- | --- | --- | --- | --- |
| ingredient ranking: C > R > V > L | 1 | C | C-L | C | YES |
| predicted ranking: C > CR > R = CV > CL = RV > V = RL > VL > L | 1 | C | C-V | C | YES |
| C = cherry | 1 | C | C-R | C | YES |
| L = diluted lemon | 1 | C | L-R | C | YES |
| R = rhubarb | 1 | C | R-V | C | YES |
| V = dilluted vinegar | 1 | C | L-V | C | YES |
| yellow highlight = key trials | 1 | L | L-R | L-R | YES |
| orange highlight = tie trials | 1 | L | R-V | R-V | YES |
|  | 1 | L | C-L | C-L | YES |
|  | 1 | L | L-V | L-V | YES |
|  | 1 | L | C-R | C-R | YES |
|  | 1 | L | C-V | C-V | YES |
|  | 1 | R | C-L | R | YES |
|  | 1 | R | C-R | R | **NO** |
|  | 1 | R | C-V | C-V | YES |
|  | 1 | R | L-R | R | YES |
|  | 1 | R | R-V | R | YES |
|  | 1 | R | L-V | R | YES |
|  | 1 | V | C-L | C-L | YES |
|  | 1 | V | C-R | C-R | YES |
|  | 1 | V | C-V | C-V | YES |
|  | 1 | V | L-R | L-R | YES |
|  | 1 | V | R-V | R-V | YES |
|  | 1 | V | L-V | L-V | YES |
|  | 2 | C | C-L | C | YES |
|  | 2 | C | C-V | C | YES |
|  | 2 | C | C-R | CR | **NO** |
|  | 2 | C | L-R | C | YES |
|  | 2 | C | R-V | C | YES |
|  | 2 | C | L-V | C | YES |
|  | 2 | L | L-R | L-R | YES |
|  | 2 | L | R-V | R-V | YES |
|  | 2 | L | C-L | C-L | YES |
|  | 2 | L | L-V | L | **NO** |
|  | 2 | L | C-R | C-R | YES |
|  | 2 | L | C-V | C-V | YES |
|  | 2 | R | C-L | R | YES |
|  | 2 | R | C-R | CR | YES |
|  | 2 | R | C-V | R | YES |
|  | 2 | R | L-R | R | YES |
|  | 2 | R | R-V | R | YES |
|  | 2 | R | L-V | R | YES |
|  | 2 | V | C-L | C-L | YES |
|  | 2 | V | C-R | C-R | YES |
|  | 2 | V | C-V | C-V | YES |
|  | 2 | V | L-R | L-R | YES |
|  | 2 | V | R-V | R-V | YES |
|  | 2 | V | L-V | L-V | YES |
